# AUBER: Automated BERT regularization

**DOI:** 10.1371/journal.pone.0253241

**Published:** 2021-06-28

**Authors:** Hyun Dong Lee, Seongmin Lee, U. Kang

**Affiliations:** 1 Columbia University, New York, NY, United States of America; 2 Seoul National University, Seoul, Republic of Korea; Taipei Medical University, TAIWAN

## Abstract

How can we effectively regularize BERT? Although BERT proves its effectiveness in various NLP tasks, it often overfits when there are only a small number of training instances. A promising direction to regularize BERT is based on pruning its attention heads with a proxy score for head importance. However, these methods are usually suboptimal since they resort to arbitrarily determined numbers of attention heads to be pruned and do not directly aim for the performance enhancement. In order to overcome such a limitation, we propose AUBER, an automated BERT regularization method, that leverages reinforcement learning to automatically prune the proper attention heads from BERT. We also minimize the model complexity and the action search space by proposing a low-dimensional state representation and dually-greedy approach for training. Experimental results show that AUBER outperforms existing pruning methods by achieving up to 9.58% better performance. In addition, the ablation study demonstrates the effectiveness of design choices for AUBER.

## Introduction

How can we effectively regularize BERT (Bidirectional Encoder Representations from Transformers) [1]? In NLP (Natural Language Processing), fine-tuning a large-scale pre-trained language model has greatly enhanced generalization. In particular, BERT has demonstrated effectiveness through improvements in many downstream NLP tasks such as sentence classification and question answering.

Despite its recent success and wide adoption, fine-tuning BERT on a downstream task is prone to overfitting due to overparameterization; BERT-base has 110M parameters and BERT-large has 340M parameters. The overfitting worsens when the target downstream task has only a small number of training examples. [1–3] show that datasets with 10,000 or less training examples sometimes fail to fine-tune BERT.

To mitigate this critical issue, multiple studies attempt to regularize BERT by pruning parameters or using dropout to decrease its model complexity [4–6]. Among these approaches, we focus on regularizing BERT by pruning attention heads since pruning yields simple and explainable results and it can be used along with other regularization methods. In order to avoid combinatorial search, whose computational complexity grows exponentially with the number of heads, the existing methods measure the importance of each attention head based on heuristics such as an approximation of sensitivity of BERT to pruning a specific attention head. However, these approaches are based on hand-crafted heuristics that are not directly related to the model performance, and therefore, would result in suboptimal performance. Moreover, all the existing methods cannot find out the optimal number of the attention heads to be pruned. Thus, the number has to be arbitrarily selected even though the optimal number significantly differs depending on the tasks.

In this paper, we propose AUBER, an effective method for regularizing BERT. AUBER overcomes the limitation of past attempts to prune attention heads from BERT by leveraging reinforcement learning. When pruning attention heads from BERT, our method automates this process by learning policies rather than relying on a rule-based policy and heuristics. Thanks to the automation, AUBER does not require us to predetermine any of the key parameters, such as the number of the attention heads to be pruned. AUBER prunes BERT sequentially in a layer-wise manner so as to avoid prohibitively large search space. For each layer, AUBER extracts features that represent the state of the layer and feeds the features to the reinforcement learning agent to determine which attention head to prune from the layer. Among numerous methods to represent the state, AUBER effectively summarizes the state of the layer into a low-dimensional vector for the sake of the scalability of the reinforcement learning agent. The final pruning policy found by the reinforcement learning agent is used to prune the corresponding layer. Before AUBER proceeds to process the next layer, BERT is fine-tuned to recapture the information lost due to pruning attention heads.

**Fig 1:**
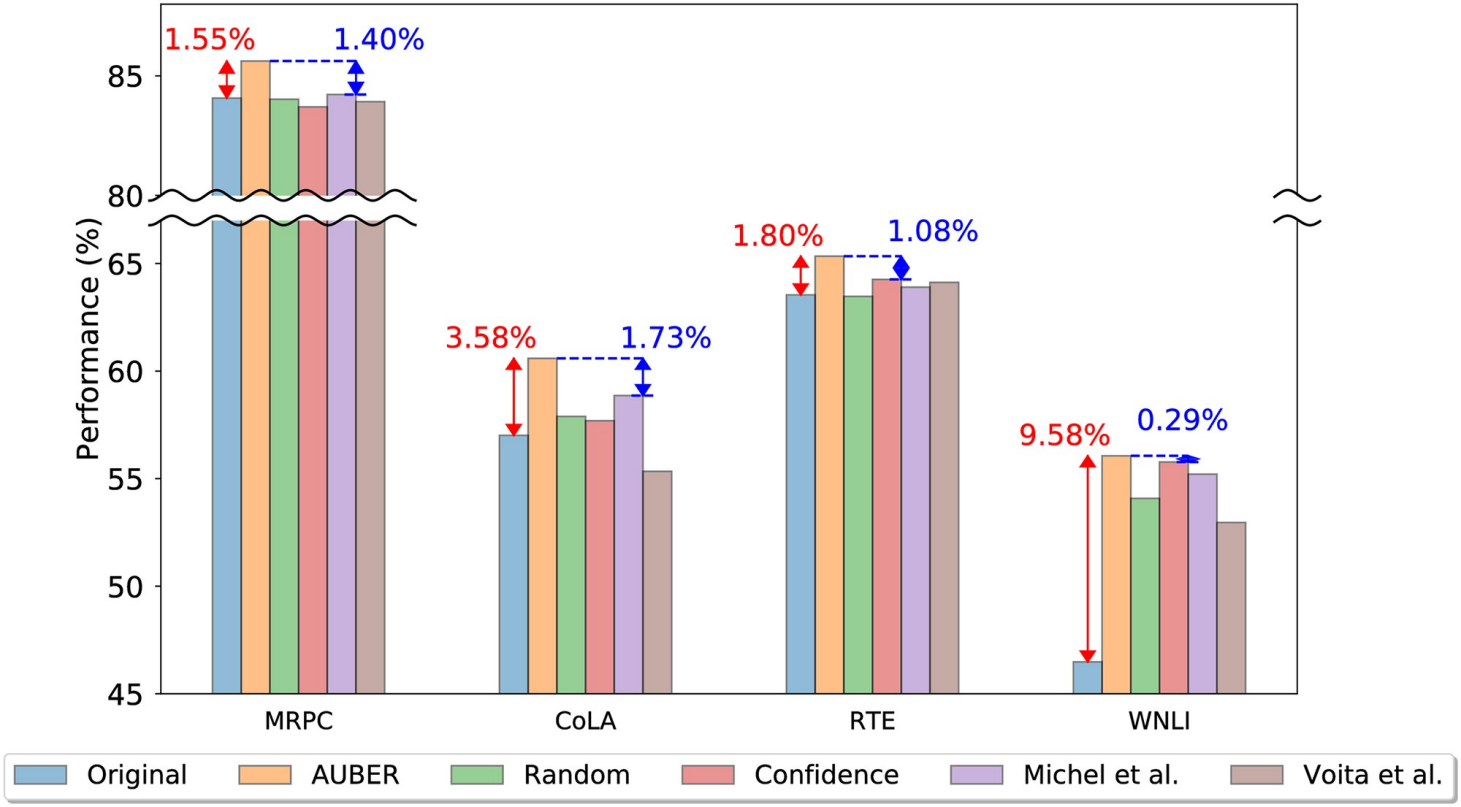
Performance of AUBER and its competitors on 4 GLUE datasets. AUBER successfully regularizes BERT model, enhancing the model performance up to 9.58%. AUBER provides the best performance among the state-of-the-art BERT attention head pruning methods.

**Fig 2:**
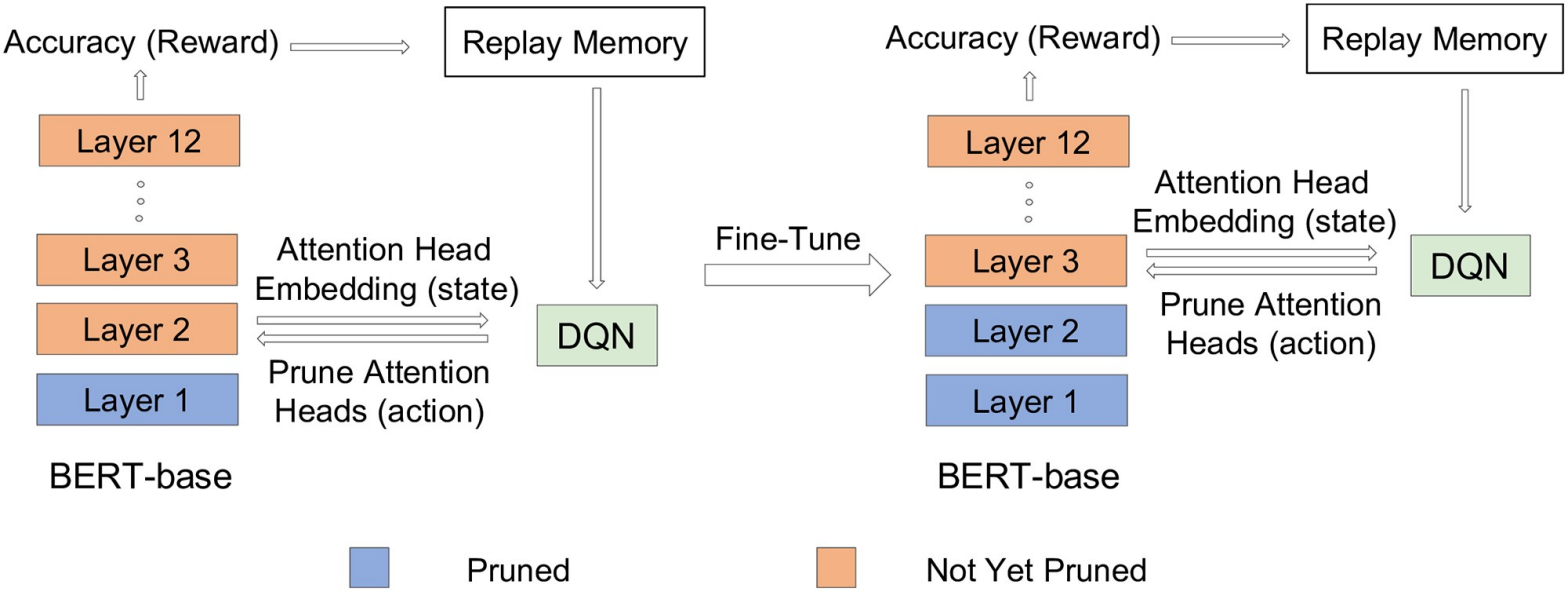
Overview of transitioning in AUBER. The figure shows the transition from *Layer* 2 to *Layer* 3 in AUBER with BERT-base.

Fig 1 shows the superiority of AUBER compared to the existing methods for pruning BERT attention head. An overview of AUBER transitioning from the second to the third layer of BERT is demonstrated in Fig 2. Our contributions are summarized as follows:

- **Method**. We propose AUBER to automatically learn how to effectively regularize BERT exploiting reinforcement learning. AUBER is designed to carefully represent the state of BERT with a low-dimensional vector and reduce the action search cost by the dually-greedy pruning, a training method we proposed for AUBER.
- **Analysis**. We theoretically justify our design choice of using the L1 norm of the value matrix of each attention head as an element of a state vector (see Theorem 1).
- **Experiments**. We perform extensive experiments and show that AUBER successfully regularizes BERT improving the performance by up to 9.58% and outperforms other head pruning methods. Through ablation studies, we empirically show that our design choices for AUBER are effective.

In the rest of this paper, we first introduce the related works and preliminaries. Then, we describe our proposed method and experimentally evaluate the performance of AUBER and its competitors. The code for AUBER can be found in https://github.com/snudatalab/AUBER.

## Related work

### BERT regularization

To prevent overfitting of BERT on downstream NLP tasks, various regularization techniques have been proposed. Variants of dropout improve the stability of fine-tuning large pre-trained language models even when presented with a small number of training examples [6, 7]. Using a slanted triangular learning rate schedule and discriminative fine-tuning has been proven to effectively prevent overfitting [8]. [9] proposes SMART, which regularizes the BERT model by smoothing it and preventing aggressive updates while [10] trains the BERT model with a multi-task learning algorithm to remedy overfitting. Introducing adversarial training to enhance the generalization of BERT has been tackled in [11]. Pre-training tasks have also been modified for better regularization. Xu et al. [12] modify the pre-training task to additionally predict previous sentences so that it can capture more correlation. Our method has definite advantages since it can be used along with any of the aforementioned methods.

### BERT pruning

A number of studies have analyzed the effectiveness of pruning parameters in BERT. [13] experimentally demonstrates overparameterization of BERT by showing that pruning 30–40% of parameters hardly affects model performance. [14] finds out the best parameter pruning strategy from the viewpoint of the lottery ticket hypothesis while [15] deploys reweighted L1 regularization with a proximal algorithm. However, these methods primarily aim to compress the BERT model not to regularize it, and, therefore, no specific method to enhance the model performance has been proposed.

Thanks to the unique structure of BERT that consists of multi-headed attention, studies on the attention heads [16] and the structured attention head pruning [4, 5, 17, 18] have been conducted as well. In [16], Kovaleva et al. reveal the information that each attention head contains through qualitative and quantitative analysis. [4, 18] evaluate the importance of each attention head by measuring heuristics such as the average of its maximum attention weight where the average is taken over tokens in a set of sentences used for evaluation, or the expected sensitivity of the model to attention head pruning. Their results show that a large percentage of attention heads with low importance scores can be pruned without significantly impacting performance. The approach to set L0 regularization term to minimize both the training loss and the number of attention heads being used has also been presented [5, 17]. However, they usually yield suboptimal results since they predetermine the order in which the attention heads are pruned by using heuristics.

### Automation of neural network pruning

To automate the process of Convolutional Neural Network pruning, [19, 20] leverage reinforcement learning to determine the best pruning strategy for each layer. Important features that characterize a layer are provided to a reinforcement learning agent to determine how much of the current layer should be pruned. To the best of our knowledge, AUBER is the first attempt to use reinforcement learning to prune attention heads from Transformer-based models such as BERT.

## Preliminaries

### Multi-headed self-attention

An attention function [21] maps a query vector and a set of key-value vector pairs to an output. We compute the query, key, and value vectors by multiplying the input embeddings $E^{Q},E^{K},E^{V}\in R^{N\times d}$ with the parameterized matrices $W^{Q}\in R^{d\times n}$, $W^{K}\in R^{d\times n}$, and $W^{V}\in R^{d\times m}$ respectively, where *N* is the number of tokens in the sentence, and *n*, *m*, and *d* are query, value, and embedding dimension respectively. In other words, *Q* = *E*^*Q*^*W*^*Q*^, *K* = *E*^*K*^*W*^*K*^, *V* = *E*^*V*^*W*^*V*^, where *Q*, *K*, and *V* are the matrices of the query, key, and value vectors respectively. In this paper, we name the matrices *W*^*Q*^, *W*^*K*^, and *W*^*V*^ as *query matrix*, *key matrix*, and *value matrix*, respectively, so as to distinguish them with *Q*, *K*, and *V*, which vary with the input data. Then, the output of the attention function is formulated as

$$\begin{array}{c} Att\left(E^{Q},E^{K},E^{V}\right)=softmax\left(\frac{QK^{⊤}}{\sqrt{n}}\right)V, \end{array}$$ (1)

where the *softmax* function is taken in a row-wise manner to compute the weighted sum of the value vectors.

In multi-headed attention, *H* independently parameterized attention heads are applied in parallel to project the input embeddings into multiple representation subspaces. Each attention head contains parameter matrices $W_{i}^{Q}\in R^{d\times n}$, $W_{i}^{K}\in R^{d\times n}$, and $W_{i}^{V}\in R^{d\times m}$, where *i* = 1, 2, …, *H*. Output matrices of *H* independent self-attention heads are concatenated and projected by a matrix $W^{O}\in R^{Hm\times d}$ to obtain the final result. This process can be represented as:

$$\begin{array}{c} MultiHeadAtt\left(E^{Q},E^{K},E^{V}\right)=Concat\left(Att_{1...H}\left(E^{Q},E^{K},E^{V}\right)\right)W^{O}, \end{array}$$ (2)

where *Att*_*i*_(*E*^*Q*^, *E*^*K*^, *E*^*V*^) is the output of the attention function with $W_{i}^{Q}$, $W_{i}^{K}$, and $W_{i}^{V}$ as the parameterized matrices.

A self-attention function follows the same mapping methods as a general attention function except that all the query, key, and value embeddings come from the same sequence. Likewise, multi-headed self-attention is a multi-headed attention function that takes the input embeddings from a common sequence.

### BERT

BERT [1] is a language representation model that has achieved state-of-the-art performance on a variety of downstream language processing tasks. Its superior performance is attributed to the well-designed pre-training techniques, the capability of considering bidirectional context, and usage of the Transformer encoder [21], which is based on multi-headed self-attention. The model consists of an embedding layer, multi-layer encoders of Transformer, and a task-dependent final fully connected layer. It is first pre-trained on masked language model and next sentence prediction tasks. It is then fine-tuned on specific tasks including language inference and question answering.

BERT-base has 12 layers of Transformer encoder blocks and each layer has 12 self-attention heads; there is a total of 144 self-attention heads in BERT-base. Despite its success in various NLP tasks, BERT sometimes overfits when the training dataset is small due to overparameterization. Thus, there has been a growing interest in BERT regularization through various methods such as dropout [6] and pruning [4, 5].

### Deep Q-learning

Deep Q Network (DQN) [22] is one of the most widely used reinforcement learning strategies to find out the optimal policy when the action space is discrete. It consists of a multi-layer neural network that takes a state *s* as an input and outputs a vector of action-value pairs for every possible action; it is a function that maps a *d*_*s*_-dimensional state space to a *d*_*a*_-dimensional action space. Here, value is the expectation of the total rewards under the consideration of a decaying factor.

Two important features of the DQN algorithm are target network and experience replay. The target network has the same architecture as that of the policy network, and its parameters are copied every *τ* steps from the policy network. It makes the training more stable by preventing the target network from being updated every step.

Experience replay introduces first-in-first-out memory buffer, replay memory, in order to resolve the existing limitations. Without the memory buffer, the training samples are obtained based only on the current state; therefore the samples have a strong correlation with each other and are dominated by the optimal action. Experience replay stores transition tuples (i.e. (state, action, reward, next state)) in the replay memory continuously, and a mini-batch randomly sampled from the memory updates the parameters of the policy network. This eliminates the detrimental correlation among the training samples and increases data efficiency by allowing each training sample to contribute to multiple parameter updates.

## Methods

We propose AUBER, our method for automatically regularizing BERT by learning the best strategy to prune attention heads from BERT. After presenting the overview of the proposed method, we describe how we frame the problem of pruning attention heads into a reinforcement learning problem. Then, we explain how states are represented in AUBER and provide a justification for the process. The next section describes how AUBER reduces the extremely large search space.

### Overview

We observe that BERT is prone to overfitting for tasks with a few training data. However, the existing head pruning methods rely on hand-crafted heuristics and hyperparameters, which give sub-optimal results. The goal of AUBER is to automate the pruning process for successful regularization. Designing such regularization method entails the following challenges:

1. **Automation**. How can we automate the head pruning process for regularization without resorting to sub-optimal heuristics and manually selected hyperparameters?
2. **Efficient and effective state representation**. When formulating the automated regularization process as a reinforcement learning problem, how can we represent the state of BERT in a way useful for pruning and tractable for training?
3. **Action search space scalability**. BERT has many parameters and attention heads. How can we handle prohibitively large action search space for pruning?

We propose the following main ideas to address the challenges:

1. **Automated regularization with reinforcement learning**. We exploit reinforcement learning, specifically DQN, with performance enhancement as a reward. DQN has shown superior performance for many tasks and is a natural choice for model-free and off-policy learning [23], which is exactly our setting. Experience replay also allows efficient usage of previous experiences and gives stable convergence [22].
2. **Low-dimensional state representation with L1 norm of the value matrix**. We represent the state of a layer with a low-dimensional vector with L1 norm of the value matrix of each attention head. We give a theoretical justification for the representation.
3. **Dually-greedy pruning**. To reduce the search space, we use two greedy methods: 1) we prune layer-by-layer, and an action is performed only within a layer, and 2) in each layer, we prune one attention head at a time and never retrieve the pruned heads to reduce the search space.

### Automated regularization with reinforcement learning

**Fig 3:**
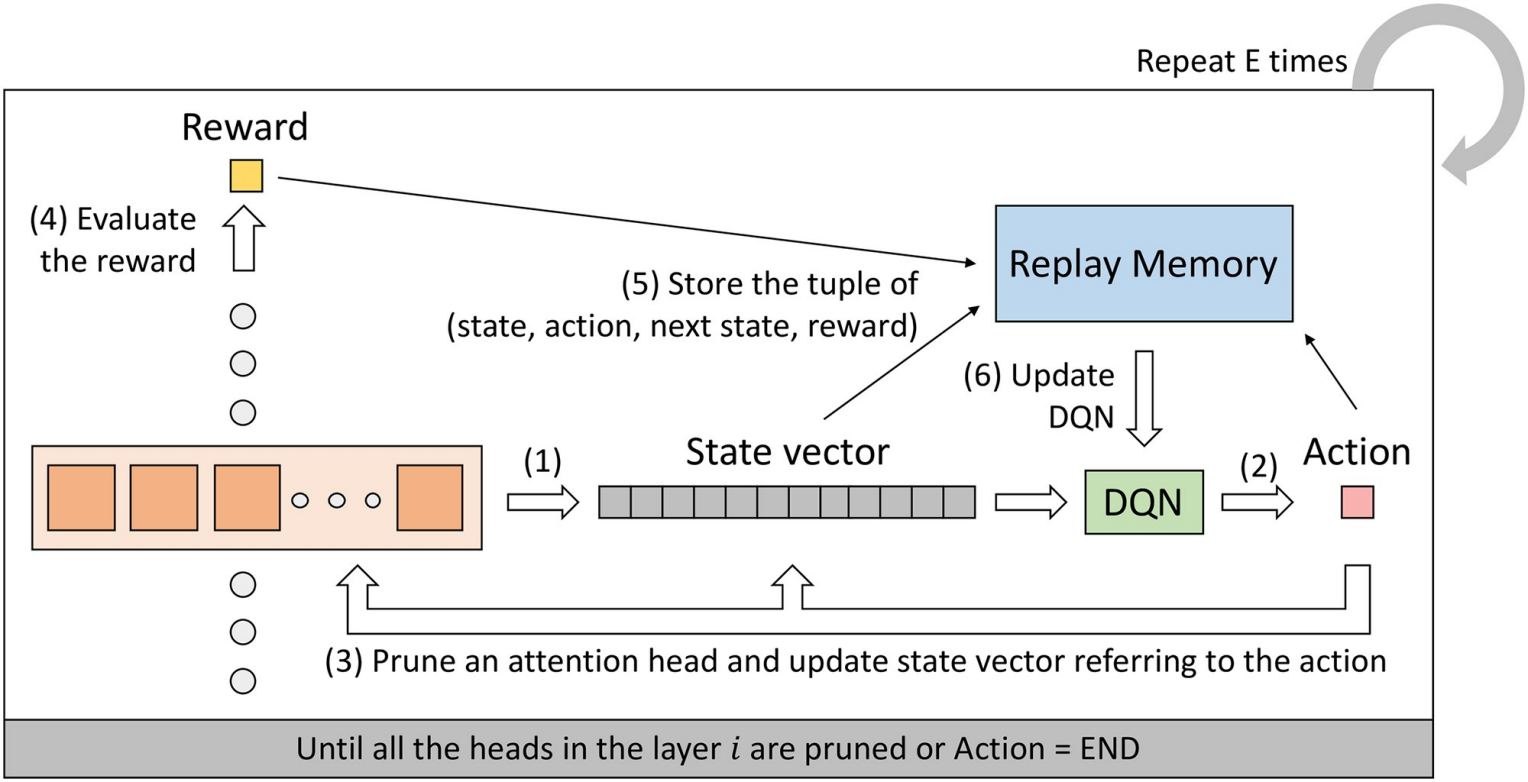
Overall flow of training AUBER on a layer. AUBER trains DQN to find out the attention heads that should be pruned for a better regularization following the illustrated steps.

AUBER leverages reinforcement learning for efficient search of regularization strategy without relying on heuristics. We exploit DQN among various reinforcement learning frameworks that have shown superior performance in model-free and off-policy environments. The overall flow is described in Fig 3. In this section, we explain the detailed settings of our reinforcement learning framework.

#### State vector

There are numerous methods to summarize the input states for DQN by deploying query, key, or value matrices, which are independent of the input data. For example, a naive approach to directly use the whole query, key, and value matrices in the current layer can represent the state of the layer. However, it gives complicated and high-dimensional state representations which result in prohibitively large DQN. Thus, we aim to obtain a concise but effective state representation and reduce the number of parameters in DQN.

#### Initial state

Each layer of BERT has multiple attention heads, each of which has its own query, key, and value matrices. For layer *l* of BERT, we derive the initial state *s*_*l*_ using L1 norm of the value matrix of each attention head. Further details for this computation is elaborated in the next section.

#### Action

The action space of AUBER is discrete. For a BERT model with *H* attention heads per layer, the number of possible actions is *H* + 1 (i.e. {1, 2, …, *H* − 1, *H*, *END*}). When the action *a* ∈ {1, 2, …, *H* − 1, *H*} is chosen, the corresponding *a*^*th*^ attention head is pruned. The action *a* = *END* signals the DQN agent to quit pruning. To facilitate exploration via off-policy learning, actions are chosen in a decaying-epsilon-greedy manner: the agent chooses a random action with the probability *ϵ* that decays over time or otherwise selects an action based on the current policy, where

$$\begin{array}{c} \epsilon =\epsilon_{final}+\frac{\epsilon_{initial}-\epsilon_{final}}{e^{n_{action}/\epsilon_{decay}}}. \end{array}$$ (3)

Here, *n*_*action*_ is the total number of actions taken by the agent up to the current episode, *ϵ*_*initial*_ is the starting value of *ϵ* (i.e. when *n*_*action*_ = 0), *ϵ*_*final*_ is the value that *ϵ* converges to as *n*_*action*_ → ∞, and *ϵ*_*decay*_ is a hyperparameter that adjusts the rate of decay of *ϵ*.

#### Next state

After the *i*^*th*^ head is pruned, the value of *i*^*th*^ index of *s*_*l*_, the *H*-dimensional state vector of the layer *l*, is set to 0. This modified state is provided as the next state to the agent. This mechanism allows the agent to recognize which attention heads have been pruned and decide the next best pruning policy based on past decisions. When the action *a* = *END*, the next state is set to the terminating state which ends an episode.

#### Reward

The reward of AUBER is the change in performance,

$$\begin{array}{c} \Delta perf=current\_performance-previous\_performance, \end{array}$$ (4)

where *current*_*performance* is the performance of the current BERT model, and *previous*_*performance* is the performance obtained from the previous state or the performance of the original BERT model when no attention heads are pruned. The performance is evaluated with the most suitable metric for each dataset.

To evaluate the reward, the training data are split into two sets: mini-training set and mini-dev set. We use the mini-dev set for the reward evaluation and the mini-training set for the fine-tuning, which will be described in the next paragraph.

If we set the reward simply as *current*_*performance*, DQN cannot capture the differences among reward values if the changes in performance are relatively small. Setting the reward as the change in performance has the normalization effect, thus stabilizing the training process of the DQN agent. The reward for action *a* = *END* is a hyperparameter that can be adjusted to encourage or discourage active pruning. In AUBER, it is set to 0 to encourage the DQN agent to prune only when the expected change in performance is positive.

#### Fine-tuning

After the best pruning policy for layer *l* of BERT is found, the BERT model pruned according to the best pruning policy is fine-tuned with a smaller learning rate. This fine-tuning step is crucial since it adjusts the weights of the remaining attention heads to compensate for the information lost due to pruning. We use the mini-training dataset for fine-tuning. When all layers are pruned by AUBER, the final model is fine-tuned with the entire training dataset with early stopping.

### State representation

In order to make DQN scalable, we summarize the state of each layer into an *H*-dimensional vector, where *H* is the number of attention heads in the layer. The initial state *s*_*l*_ of layer *l* of BERT is computed through the following procedure. We first calculate the L1 norm of the value matrix of each attention head in the layer *l*. Then, we standardize the norm values to have a mean *μ* = 0 and a standard deviation *σ* = 1. Finally, the *softmax* function is applied to the norm values to yield *s*_*l*_. The justification of using the L1 norm of the value matrix is given by Theorem 1 which states that the L1 norm of the value matrix of a head upper bounds the L1 norm of its output matrix, which implies the importance of the head in the layer.

**Theorem 1**. *For a layer with H heads, let N be the number of tokens in the sentence and m*, *n*, *and d be the value, query, and embedding dimension respectively. Let* $E^{Q},E^{K},E^{V}\in R^{N\times d}$ *be the input query, key, and value embedding matrices, and* $W_{i}^{Q}\in R^{d\times n}$, $W_{i}^{K}\in R^{d\times n}$, *and* $W_{i}^{V}\in R^{d\times m}$ *be the query, key, and value matrices of the i*^*th*^*head. Let O*_*i*_ *be the output of the i*^*th*^*head. Then*, ${∥O_{i}∥}_{1}\leq C{∥W_{i}^{V}∥}_{1}$, *where the constant C* = *N*‖*E*^*V*^‖_1_ *and the norm of the matrices is entrywise norm*: ‖*A*‖_1_ = ∑_*j*_ ∑_*k*_ |*A*_*jk*_|.

*Proof*. For *i*^*th*^ head in the layer, let

$$\begin{array}{c} softmax_{i}=softmax\left(\frac{\left(E^{Q}W_{i}^{Q}\right){\left(E^{K}W_{i}^{K}\right)}^{⊤}}{\sqrt{n}}\right) \end{array}$$ (5)

and

$$\begin{array}{c} v_{i}=E^{V}W_{i}^{V}. \end{array}$$ (6)

The output of the *i*^th^ head, *O*_*i*_, is evaluated as *O*_*i*_ = *softmax*_*i*_ *v*_*i*_. Then,

$${∥O_{i}∥}_{1}=\sum_{j=1}^{N}\sum_{k=1}^{m}\left|{\left(O_{i}\right)}_{jk}\right|$$ (7)

$$\begin{array}{cc} & =\sum_{j=1}^{N}\sum_{k=1}^{m}|{\left({\left(softmax_{i}\right)}_{j\cdot }\right)}^{⊤}{\left(v_{i}\right)}_{\cdot k}| \end{array}$$ (8)

$$\begin{array}{cc} & \leq \sum_{j=1}^{N}\sum_{k=1}^{m}{∥{\left(softmax_{i}\right)}_{j\cdot }∥}_{2}{∥{\left(v_{i}\right)}_{\cdot k}∥}_{2} \end{array}$$ (9)

$$\begin{array}{cc} & =\sum_{j=1}^{N}{∥{\left(softmax_{i}\right)}_{j\cdot }∥}_{2}\sum_{k=1}^{m}{∥{\left(v_{i}\right)}_{\cdot k}∥}_{2} \end{array}$$ (10)

Since the L1 norm of a vector is always greater than or equal to the L2 norm of the vector,

$${∥O_{i}∥}_{1}\leq \sum_{j=1}^{N}{∥{\left(softmax_{i}\right)}_{j\cdot }∥}_{1}\sum_{k=1}^{m}{∥{\left(v_{i}\right)}_{\cdot k}∥}_{1}$$ (11)

$$\begin{array}{cc} & =N\sum_{k=1}^{m}∥{\left(v_{i}\right)}_{\cdot k}{∥}_{1} \end{array}$$ (12)

$$\begin{array}{cc} & =N\sum_{j=1}^{N}\sum_{k=1}^{m}\left|{\left(v_{i}\right)}_{jk}\right| \end{array}$$ (13)

$$\begin{array}{cc} & =N\sum_{j=1}^{N}\sum_{k=1}^{m}\left|{\left(E_{j\cdot }^{V}\right)}^{⊤}{\left(W_{i}^{V}\right)}_{\cdot k}\right| \end{array}$$ (14)

$$\begin{array}{cc} & \leq N\sum_{j=1}^{N}\sum_{k=1}^{m}{∥E_{j\cdot }^{V}∥}_{2}{∥{\left(W_{i}^{V}\right)}_{\cdot k}∥}_{2} \end{array}$$ (15)

$$\begin{array}{cc} & =N\sum_{j=1}^{N}{∥E_{j\cdot }^{V}∥}_{2}\sum_{k=1}^{m}{∥{\left(W_{i}^{V}\right)}_{\cdot k}∥}_{2} \end{array}$$ (16)

$$\begin{array}{cc} & \leq N\sum_{j=1}^{N}{∥E_{j\cdot }^{V}∥}_{1}\sum_{k=1}^{m}{∥{\left(W_{i}^{V}\right)}_{\cdot k}∥}_{1} \end{array}$$ (17)

$$\begin{array}{cc} & =N{∥E^{V}∥}_{1}{∥W_{i}^{V}∥}_{1}. \end{array}$$ (18)

All heads in the same layer take the same *E*^*V*^ as input and *N* is constant. Thus,

$$\begin{array}{c} {∥O_{i}∥}_{1}\leq C{∥W_{i}^{V}∥}_{1} \end{array}$$ (19)

for the constant *C* = *N*‖*E*^*V*^‖_1_.

Theorem 1 implies that the importance of the *i*^th^ attention head in its layer is bounded by the L1 norm of the head’s value matrix $W_{i}^{V}$. Therefore, the L1 norm of the value matrix in each head can be exploited to represent the state of the layer.

### Dually-greedy search space pruning

The total number of attention heads in BERT-base is 144 as it consists of 12 layers each of which has 12 attention heads. Naively designing actions would lead to 2^144^ possible actions which are prohibitively large. Our idea to reduce the search space is dually-greedy pruning: we prune layer-by-layer in a greedy manner (from lower to upper layers), and in each layer, we greedily prune one single attention head at a time.

For each layer *l* with *H* attention heads, the DQN agent receives an initial layer embedding *s*_*l*_ which encodes useful characteristics (L1 norm of the value matrix in each attention head) of this layer. Then, the agent outputs the index of an attention head that is expected to increase the training performance when removed. After an attention head *i* is pruned, the value of the *i*^*th*^ index of *s*_*l*_ is set to 0, and it is provided as the next state to the agent. This process is repeated until the action *a* becomes *END*, which means pruning more heads would deteriorate the model performance. The model pruned up to layer *l* is fine-tuned on the mini-training dataset, and a new initial layer embedding *s*_*l*+1_ is calculated from the fine-tuned model.

Algorithm 1 illustrates the process of AUBER. AUBER receives a BERT model *B*_*t*_, which is fine-tuned on a specific task *t*, the parameters *L* and *H* for BERT, and the number *E* of reinforcement learning episodes. AUBER aims to output the regularized *B*_*t*_. Lines 2-30 are conducted in a layer-wise manner. In line 2, we initialize a policy network *P* and a replay memory *M* for layer *l*. Lines 3-28 train the policy network *P* for *E* episodes. For each episode, lines 8-25 choose an *action* based on the current state vector *s*_*l*_, prune an attention head based on the *action*, compute the resulting *reward* and the next state $s_{l}^{*}$, and store the transition tuple into the memory *M*. This process is repeated until *action* = *END*, which indicates the termination of pruning. In line 27, we optimize *P* with transition tuples sampled from *M*. After the training of the policy network is finished, in lines 29-30, we use the trained policy network to find the optimal pruning policy for layer *l*, prune *B*_*t*_ according to the policy, and finally fine-tune *B*_*t*_. After pruning a layer *l*, we proceed to prune the next layer *l* + 1 up to the final layer *L*.

**Algorithm 1**: AUBER: Automated BERT regularization

**Input**: A BERT model *B*_*t*_ fine-tuned on task *t*, # *L* of layers in BERT model, # *H* of attention heads per layer of BERT model, and # *E* of episodes.

**Output**: Regularized *B*_*t*_.

1 **for** *l* ← 1 **to** *L* **do**

2  Initialize policy network *P* and replay memory *M*

3  **for** *e* ← 1 **to** *E* **do**

4   $B_{t}^{*}\leftarrow copy\left(B_{t}\right)$

5   $s_{l}\leftarrow B_{t}^{*}.state\_vector\left(l\right)$

6   $previous\_performance\leftarrow eval(B_{t}^{*})$

7   **while** *action* ≠ *END* **do**

8    **if** $B_{t}^{*}.one\_head\_left$ **then**

9     *action* ← *END*

10    **else**

11     *action* ← *P*.*choose*_*action*(*s*_*l*_)

12    **end**

13    **if** *action* = *END* **then**

14     $s_{l}^{*}\leftarrow Terminal\;State$

15     *reward* ← 0

16    **else**

17     $B_{t}^{*}.prune\_head\left(action\right)$

18     $s_{l}^{*}\leftarrow copy\left(s_{l}\right)$

19     $s_{l}^{*}\left[action\right]\leftarrow 0$

20     $current\_performance\leftarrow eval(B_{t}^{*})$

21     *reward* ← *current*_*performance*–*previous*_*performance*

22     *previous*_*performance* ← *current*_*performance*

23    **end**

24    $M.push(s_{l},action,s_{l}^{*},reward)$

25    $s_{l}\leftarrow s_{l}^{*}$

26   **end**

27   *P*.*optimize*(*M*)

28  **end**

29  *B*_*t*_.*prune*(*P*.*final*_*policy*(*l*))

30  *B*_*t*_.*finetune*()

31 **end**

## Experiments

We conduct experiments to answer the following questions of AUBER.

**Q1 Performance**. Given a BERT model fine-tuned on a specific NLP task, how well does AUBER improve the performance of the model?
**Q2 State Representation**. How useful is the *L1 norm of the value matrices* of attention heads in representing the state of BERT?
**Q3 Order of Processing Layers**. How does the order in which the layers are processed by AUBER affect regularization?
**Q4 Performance during Pruning**. How does the performance change after the regularization of each layer is done?

### Experimental setup

#### Datasets

We test AUBER on four GLUE datasets [24]—MRPC [25], CoLA [26], RTE, and WNLI, each of which contains less than 10,000 training instances; it has been observed that datasets with 10,000 or less training examples often fail in fine-tuning BERT [1, 2]. Detailed information on the datasets is described in Table 1.

**Table 1:** Summary of the four GLUE datasets used in the experiments.

Dataset	# of classes	# of train	# of dev	Metrics
MRPC	2	3668	408	Accuracy
CoLA	2	8551	1043	Matthews
RTE	2	2490	277	Accuracy
WNLI	2	635	71	Accuracy

The URLs for the datasets are as follows: MRPC (https://www.microsoft.com/en-us/download/details.aspx?id=52398), CoLA (https://nyu-mll.github.io/CoLA/), RTE (https://aclweb.org/aclwiki/Recognizing_Textual_Entailment), and WNLI (https://cs.nyu.edu/faculty/davise/papers/WinogradSchemas/WS.html). We evaluate the performance by using *accuracy* for MRPC, RTE, and WNLI, and *Matthews correlation coefficient* for CoLA.

#### BERT model

We use the pre-trained *bert-base-cased* model with 12 layers and 12 attention heads per layer provided by huggingface (https://github.com/huggingface/transformers). We fine-tune this model on each dataset mentioned in Table 1 to obtain the initial model. Initial models for MRPC, CoLA, and WNLI are fine-tuned on the corresponding dataset for 3 epochs, and that for RTE is fine-tuned for 4 epochs. The maximum sequence length is set to 128, and the mini-batch size per GPU is set to 32. The learning rate for fine-tuning initial models for MRPC, CoLA, and WNLI is set to 0.00002, and that for RTE is set to 0.00001. We denote the initial models as *Original* and report the accuracies in Table 2.

**Table 2:** Performance of AUBER and its competitors. AUBER gives the best performance for the same number of pruned heads. Bold font indicates the best performance among competing pruning methods.

	MRPC	CoLA	RTE	WNLI
Original	84.07	57.01	63.54	46.48
AUBER	**85.62±0.51**	**60.59±0.73**	**65.34±1.30**	**56.06±0.63**
Random	84.02±1.12	57.89±0.90	63.47±1.29	54.08±2.14
Confidence	83.70±0.47	57.69±2.19	64.26±1.64	55.77±0.77
Michel et al. [4]	84.22±0.33	58.86±0.64	63.90±0.00	55.21±1.84
Voita et al. [5]	83.92±0.71	55.34±0.81	64.12±1.65	52.96±5.51

#### Reinforcement learning

We use a 4-layer feedforward neural network for the DQN agent. The input dimension is 12 and the output dimension is 13. The dimension of all hidden layers is set to 512. LeakyReLU is applied after all layers except for the last one. We train the DQN agent for 150 episodes. For the epsilon greedy strategy to choose actions, the initial epsilon value *ϵ*_*initial*_ and final epsilon value *ϵ*_*final*_ are set to 1 and 0.05 respectively, and the epsilon decreases exponentially with the decay rate *ϵ*_*decay*_ of 256. The replay memory size is set to 5000, and the batch size for training the DQN agent is set to 128. The discount value *γ* for the DQN agent is set to 1. The learning rate is set to 0.000002 when fine-tuning BERT after processing a layer. Before processing each layer, the training dataset is randomly split into 1: 2 to yield a mini-training dataset and a mini-dev dataset. When fine-tuning the final model, the patience value of early stopping is set to 20.

#### Competitors

We compare AUBER with other methods that prune BERT’s attention heads. If AUBER prunes *P* number of attention heads from BERT, we prune *P* heads in all the competitors. To be fair, we conduct attention head pruning and fine-tuning in a layer-wise manner also for the competitors.

- **Random**. Prune attention heads randomly.
- **Confidence**. Prune *P* heads with the smallest confidence score, which is the average of the maximum attention weight after a series of forward passes. A high confidence score indicates that the weight is concentrated on a single token.
- **Michel et al**. [4]. Perform a forward and backward pass to calculate gradients and use them to assign an importance score to each attention head.
- **Voita et al**. [5]. Construct a new loss function that minimizes both the classification error and the number of used heads so that unproductive heads are pruned while maintaining the model performance.

#### Implementation

We construct all models using the PyTorch framework. All the models are trained and tested on a GeForce GTX 1080 Ti GPU.

### Performance

We evaluate the performance of AUBER against competitors. We repeat the experiments five times and report the average and the standard deviation of the performance. Table 2 shows the results on four GLUE datasets listed in Table 1. Note that AUBER outperforms all of its competitors on regularizing BERT, providing the best performance for all the datasets. While most of the competitors fail to improve the performance of BERT on the dev dataset of MRPC and CoLA, AUBER improves the performance of BERT by up to 9.58% on those datasets. The consistent enhancement in all of the four representative tasks demonstrates the superiority of AUBER. AUBER’s superiority is attributable to its training method: AUBER is trained to enhance the performance, while the others aim to give the minimal influence to the performance and thus not to degrade the performance.

### Effect of state representation

We empirically demonstrate the effectiveness of our design choices for AUBER. More specifically, we validate that the *L1 norm of value matrix* of each attention head effectively guides AUBER to predict the best action. Table 3 shows the performances of the variants of AUBER.

**Table 3:** Performance of AUBER and its variants. Comparison of AUBER with four variants: AUBER-Query, AUBER-Key, AUBER-L2, and AUBER-Reverse on four GLUE datasets to demonstrate the effectiveness of various ways to calculate the initial state. AUBER-Query and AUBER-Key use the query and key matrices respectively, and AUBER-L2 uses the L2 norm of the value matrix to obtain the initial state. AUBER-Reverse processes BERT starting from the final layer (e.g. 12^th^ layer for BERT-base). Bold font indicates the best performance among pruning methods.

	MRPC	CoLA	RTE	WNLI
AUBER	**85.62±0.51**	**60.59±0.73**	**65.34±1.30**	**56.06±0.63**
AUBER-Query	83.87±0.84	55.81±0.84	65.05±1.06	47.61±5.12
AUBER-Key	83.68±0.75	56.90±1.46	63.83±0.39	50.14±7.56
AUBER-L2	82.90±1.39	57.46±1.97	64.55±1.74	40.28±12.7
AUBER-Reverse	84.56±1.39	58.07±1.27	62.24±1.43	43.67±8.15

#### AUBER with the key/query matrices as the state vector

Among the query, key, and value matrices of each attention head, we show that the value matrix best represents the current state of BERT. We evaluate the performance of AUBER against AUBER-Query and AUBER-Key. AUBER-Query and AUBER-Key use the query and key matrices respectively to obtain the initial state. Table 3 shows the performances of AUBER, AUBER-Query, and AUBER-Key on the four GLUE datasets listed in Table 1. Note that AUBER, which uses the value matrix to obtain state vectors, outperforms AUBER-Query and AUBER-Key on all four tasks.

#### AUBER with L2 norm of the value matrices as the state vector

AUBER uses the L1 norm of the value matrices to compute the state vector based on the theoretical derivation. In this ablation study, we experimentally show that the L1 norm of the value matrices is appropriate for the state vector. We set a new variant AUBER-L2, which leverages the L2 norm of the value matrices to compute the initial state vector instead of the L1 norm. The performance of AUBER is far more superior than AUBER-L2 in most cases bolstering that the L1 norm of the value matrices effectively represents the state of BERT.

### Effect of order of processing layers

We empirically demonstrate how the order in which the layers are processed affects the final performance. We evaluate the performance of AUBER against AUBER-Reverse which processes BERT layers in the opposite direction (i.e. starting from the 12^th^ layer) to what AUBER does. As shown in Table 3, AUBER provides better performance than AUBER-Reverse in every case. This shows that pruning lower layers first and then moving to upper layers by AUBER is effective. A possible explanation is that it is easier to train task-specific parameters (those in upper layers) after learning general parameters (those in lower layers), rather than that in the reverse order.

### Performance change during the pruning process

**Fig 4:**
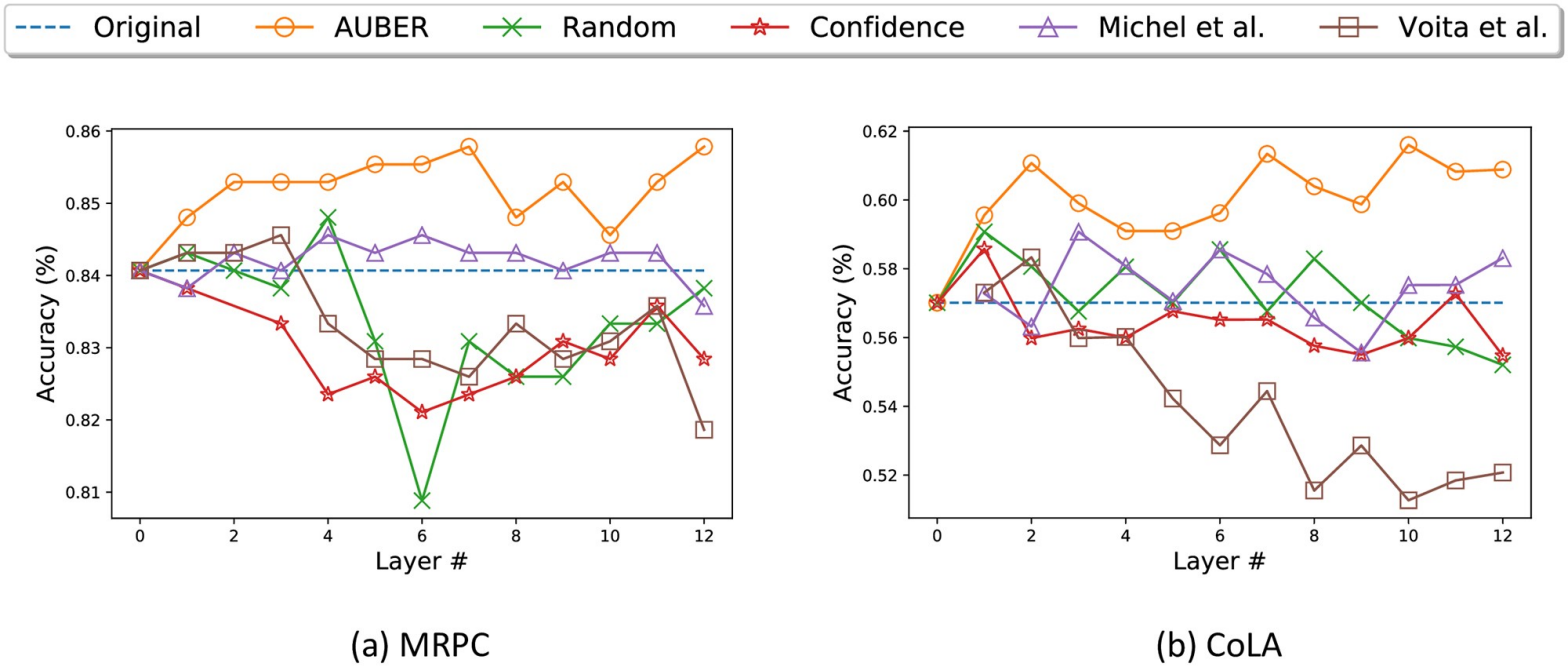
Performance after pruning the attention heads from each layer. AUBER consistently improves the model performance and achieves outstanding final performance, while all the other methods fail to enhance the model performance.

We visualize how the model performance changes as each layer is processed by AUBER and four competitors in Fig 4. We conduct the experiments with the MRPC dataset and pruning is conducted successively from Layer 1 to Layer 12; for every method, 20 attention heads are pruned. We measure the model performance after pruning the selected heads of each layer and fine-tuning the model. It shows that AUBER mostly enhances the model performance after pruning the heads in each layer, whereas all the competitors prune inappropriate attention heads and result in performance degradation. It is notable that the performance in AUBER never goes below the original performance, even in the 8^th^ and the 10^th^ layer in which performance degradation has occurred. This proves that AUBER does not prune the very important heads that can bring significant performance drop when pruned.

## Conclusion

We propose AUBER, an effective method to regularize BERT by automatically pruning attention heads. Instead of depending on heuristics or rule-based policies, AUBER leverages reinforcement learning to learn a pruning policy that determines which attention heads should be pruned for better regularization. Experimental results demonstrate that AUBER effectively regularizes BERT, increasing the performance of the original model on the dev dataset by up to 9.58%. In addition, we experimentally demonstrate the effectiveness of our design choices for AUBER.
